# MYBL2 (B-Myb): a central regulator of cell proliferation, cell survival and differentiation involved in tumorigenesis

**DOI:** 10.1038/cddis.2017.244

**Published:** 2017-06-22

**Authors:** Julian Musa, Marie-Ming Aynaud, Olivier Mirabeau, Olivier Delattre, Thomas GP Grünewald

**Affiliations:** 1 Max-Eder Research Group for Pediatric Sarcoma Biology, Institute for Pathology of the LMU Munich, Munich, Germany; 2 INSERM Unit 830 ‘Genetics and Biology of Cancers’, Institut Curie Research Center, Paris, France; 3 German Cancer Consortium (DKTK), Munich, Germany; 4 German Cancer Research Center (DKFZ), Heidelberg, Germany

## Abstract

Limitless cell proliferation, evasion from apoptosis, dedifferentiation, metastatic spread and therapy resistance: all these properties of a cancer cell contribute to its malignant phenotype and affect patient outcome. MYBL2 (alias B-Myb) is a transcription factor of the MYB transcription factor family and a physiological regulator of cell cycle progression, cell survival and cell differentiation. When deregulated in cancer cells, MYBL2 mediates the deregulation of these properties. In fact, MYBL2 is overexpressed and associated with poor patient outcome in numerous cancer entities. MYBL2 and players of its downstream transcriptional network can be used as prognostic and/or predictive biomarkers as well as potential therapeutic targets to offer less toxic and more specific anti-cancer therapies in future. In this review, we summarize current knowledge on the physiological roles of MYBL2 and highlight the impact of its deregulation on cancer initiation and progression.

## Facts

- MYBL2 is a highly conserved member of the MYB family of transcription factors.
- MYBL2 is an important physiological regulator of cell cycle progression, cell survival and cell differentiation.
- Deregulation of MYBL2 expression is involved in cancer initiation and progression.
- High MYBL2 expression is significantly correlated with poor patient outcome in numerous cancer entities.

## Open questions

- What are further players of the MYBL2 downstream transcriptional network mediating its cancer-promoting properties?
- How can MYBL2 and players of its downstream transcriptional network be exploited as therapeutic targets to improve patient outcome?
- Which additional cancer entities are also affected by MYBL2 deregulation and which patients could specifically benefit from using MYBL2 as a biomarker or therapeutic target?

Limitless replicative potential, evading apoptosis, tissue invasion and metastasis: these classical hallmarks of cancer, as originally proposed by Hanahan and Weinberg,^1^ characterize the malignant phenotype of a cancer cell. MYBL2 (V-Myb avian myeloblastosis viral oncogene homolog-like 2), a transcription factor of the MYB family of transcription factors, contributes to these properties of a cancer cell. MYBL2 is a physiological regulator of cell cycle progression, cell survival and cell differentiation, but due to its frequently found deregulation in cancer, it significantly drives cancer initiation and/or progression.

The MYB family of transcription factors comprises three members: MYB (c-Myb), MYBL1 (A-Myb) and MYBL2 (B-Myb). *MYB* was the first discovered family member and is the mammalian homolog of the retroviral v-Myb oncogene that causes acute leukemia in birds and can transform hematopoietic cells.^2, 3^ *MYBL1* and *MYBL2* have been cloned based on the homology to *MYB*.^4^ In mammals, MYB expression is mainly restricted to hematopoietic cells, colonic crypts and brain,^5, 6^ whereas MYBL1 is expressed in several regions of the developing central nervous system, germinal B-lymphocytes and reproductive systems of both genders.^7, 8^ In contrast, MYBL2 is expressed in basically all proliferating cells,^3^ which is a possible explanation for the lethal phenotype of *MYBL2* knockout mice showing early embryonal death as a result of impaired inner cell mass formation,^9^ whereas *MYBL1* deletion results in viable mice and *MYB* deletion leads to late embryonal death by cause of lacking erythropoiesis.^7, 10^

According to their tissue-specific expression, MYB and MYBL1 deregulations have been associated with certain specific cancer entities: MYB was shown to be involved in several types of leukemia, colon and breast cancer,^11^ whereas MYBL1 has been associated with Burkitt’s lymphoma and several types of leukemia.^12^ In contrast, MYBL2 deregulations occur in a broad spectrum of cancer entities as it is a central regulator of cell cycle progression, cell survival and cell differentiation in many tissue types (see ‘MYBL2 in cancer’ section). In this review, we summarize the physiological roles of MYBL2 in cell cycle regulation, cell survival and cell differentiation, and describe its deregulation as well as the resulting functional and clinical implications in cancer.

## MYBL2 in Cell Cycle Regulation

*MYBL2* is a cell cycle regulated and a cell cycle regulating gene. Its expression is controlled by the DREAM multiprotein complex (**D**imerization partner, **R**B-like proteins, **E**2Fs **a**nd **M**uvB core), which is crucial in coordinating cell cycle-dependent gene expression and represses most cell cycle genes during cellular quiescence.^13^ This complex consists of the dimerization partner (DP1, -2, -3), the RB-like proteins p130 or p107, E2F (E2F4 or E2F5) and the multi-vulval class B core (MuvB, itself consisting of LIN9, LIN37, LIN52, LIN54 and RBBP4).^13^ Upon cell cycle entry, p130 or p107 dissociate from the MuvB core and from repressor E2Fs (E2F4, E2F5) due to loss of DYRK1A-dependent phosphorylation of LIN52, allowing activator E2Fs (E2F1 or E2F2 or E2F3) to transactivate early G1/S cell cycle genes, including *MYBL2*.^13^ Accordingly, MYBL2 is repressed by the DREAM complex during cellular quiescence and becomes subsequently expressed in late G1 and in S phase.^13^ Additionally, at a post-transcriptional level, MYBL2 expression can be suppressed by microRNAs.^14, 15, 16, 17, 18, 19^

Apart from MYBL2 expression, the transcriptional activity of MYBL2 is regulated by posttranslational modifications and protein–protein interactions. During late G1 and S phase, MYBL2 is phosphorylated by Cyclin A/E-CDK2, which enhances its transactivation activity, probably by releasing it from the nuclear receptor co-repressors N-CoR and SMRT, which maintain MYBL2 in an inhibited state when non-phosphorylated.^20, 21, 22^ Interestingly, Cyclin A-dependent phosphorylation simultaneously reduces MYBL2 protein expression by facilitating ubiquitin-mediated proteolysis of MYBL2, probably to regulate the proper alternation of events during cell cycle progression.^23^ p300, a transcriptional co-activating protein, is also able to stimulate MYBL2 activity by MYBL2 acetylation, depending on its phosphorylation by Cyclin A.^24^ However, contrary to Cyclin A, Cyclin D1 strongly inhibits transactivation activity of MYBL2 through direct interaction.^25^ Mechanistically, it was proposed that Cyclin D1 abolishes the activating function of p300.^24^ Several other co-activators, such as PARP1, ZPR9, TAF(II)250, or co-repressors, such as p107, p57 or CDK9, were further shown to modulate transactivation properties of MYBL2.^26, 27, 28, 29, 30, 31, 32, 33^

**Figure 1:**
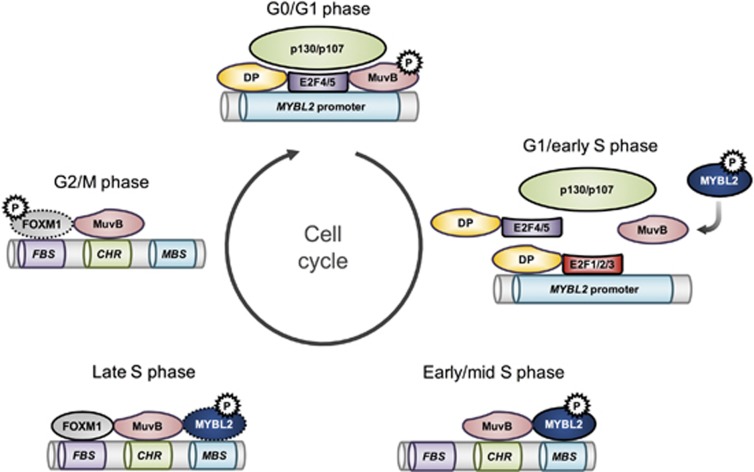
Regulation of MYBL2 expression and subsequent association of MYBL2 with its functional binding partners MuvB and FOXM1 during the cell cycle. In G0/G1 phase, the DREAM complex binds the *MYBL2* promoter and suppresses MYBL2 expression. In late G1/early S phase, the DREAM complex dissociates due to loss of DYRK1A-dependent phosphorylation of LIN52 (part of the MuvB core), MYBL2 becomes increasingly expressed (and activated by Cyclin A/E-CDK2-dependent phosphorylation) and associates with the MuvB core to cooperatively transactivate G2/M genes in early and mid S phase. In late S phase, FOXM1 additionally associates with the MuvB–MYBL2 complex and cooperates in transactivation of these late cell cycle genes. MYBL2 increasingly becomes degraded in late S phase, leading to predominantly persisting FOXM1–MuvB complexes during G2/M phase, whereby FOXM1 is increasingly activated by phosphorylation. The residual MuvB–FOXM1 complex dissociates due to increasing APC/C-CDH1-dependent FOXM1 degradation during M phase

Early results already showed that MYBL2, when expressed and activated in late G1 and S phase, directly binds to the promoters and transactivates genes expressed in G2/M phase, such as *Cyclin B1* (*CCNB1*),^34^ *CDK1*,^34^ *Cyclin A2* (*CCNA2*)^34^ (a list of selected MYBL2 target genes^34, 35, 36, 37, 38, 39, 40, 41, 42, 43, 44, 45, 46, 47, 48, 49, 50, 51, 52, 53^ is given in Table 1). Recently it was shown that the MuvB core, which dissociates from the DREAM complex upon cell cycle entry, and FOXM1 cooperate with MYBL2 to transactivate these late cell cycle genes.^13, 36^ Accordingly, MYB binding sites (MBS), cell cycle genes homology region (CHR, bound by LIN54 of the MuvB core) elements and FOXM1 binding motifs co-occur in the promoters of these genes.^13, 36^ A model emerged over the years: When DREAM dissociates upon cell cycle entry, MYBL2 becomes increasingly expressed and activated, and forms a complex with MuvB in early and mid S phase. RNAi experiments showed that knockdown of either MYBL2 or components of the MuvB core complex inhibits target gene promoter binding of the other.^36^ Consistently, depletion of either MBS or CHR motifs in the promoters of these target genes independently prohibits target gene promoter binding.^49, 54^ These results clearly indicate a dependency of both factors to one another in transactivating late cell cycle genes in early and mid S phase. Afterwards, the MYBL2–MuvB complex recruits FOXM1 in late S phase, forming a MYBL2–MuvB–FOXM1 complex.^13^ MYBL2 increasingly undergoes phosphorylation-dependent proteasomal degradation in late S phase,^23^ leading to predominant MuvB–FOXM1 complex in G2/M phase, whereby FOXM1 is increasingly activated by phosphorylation.^55^ Depletion of FOXM1 did not affect MYBL2 target gene promoter binding, but conversely, MYBL2 or LIN9 depletion reduced FOXM1 target gene promoter binding, indicating that the MYBL2–MuvB complex is required for FOXM1 target gene promoter binding, but not vice versa.^36^ As the FOXM1 DNA-binding domain has an extraordinarily low binding affinity to its target sequence and MBS and CHR motifs are necessary for FOXM1 to bind the target promoters, it was proposed that the MYBL2–MuvB complex is needed to increase target specificity for FOXM1 binding.^13^ The residual MuvB–FOXM1 complex dissociates due to increasing APC/C-CDH1-dependent FOXM1 degradation during M phase^56^ (Figure 1).

The association between MYBL2 and cell proliferation has already been described early by Arsura *et al.*^57^ Although some of the pioneering studies indicated a role for MYBL2 in G1/S progression,^58, 59^ the major role of MYBL2 in G2/M progression became increasingly clear over the recent years: RNAi-mediated MYBL2 knockdown in human cell lines and experiments in *Drosophila* with knockout of the *MYBL2 Drosophila* homolog *dMyb* reduces cell proliferation, expression of G2/M genes and decreases the amount of cells in G2/M phase.^60, 61, 37, 38, 53, 62, 63^ Although *dMyb* is the only gene of the MYB transcription factor family in *Drosophila*, it is functionally and phylogenetically equivalent to vertebrate *MYBL2* and can therefore be seen as a suitable model.^64^ The results from *Drosophila* experiments are remarkable, as they indicate that an adequate proliferative capacity mediated by MYBL2 is necessary to maintain genomic stability.^53, 65, 66, 67^ Loss-of-function mutation of *dMyb* causes abnormal mitoses that are associated with multiple functional centrosomes, unequal chromosome segregation, micronuclei formation and failure to complete cell division.^65^ These are frequent in the later cell cycles with resulting nuclei that often show aneuploidy and/or polyploidy.^65^ It was also shown that MYBL2 can contribute to genomic stability by forming complexes with Clathrin and Filamin.^68^ This is required for proper localization of Clathrin at the mitotic spindle and is thereby stabilizing kinetochore fibers.^68^ Consistently, in embryonic stem cells (ESC) MYBL2 depletion leads to stalling of replication forks, disorganization of the replication program and an increase in double-strand breaks.^41^ It has been shown that these effects are, at least in part, mediated by deregulation of *MYC* and *FOXM1* transcription, which are important for normal S phase progression, indicating that MYBL2 protects cells from genomic damage during S phase by promoting proper cell cycle progression.^41^ Chromosomal fragmentation, shorter and thicker chromatids, end-to-end fusion and chromatid loss upon MYBL2 knockdown indicates that reduced activity of MYBL2 is also associated with structural chromosomal instability.^69^

## MYBL2 in Cell Survival

**Figure 2:**
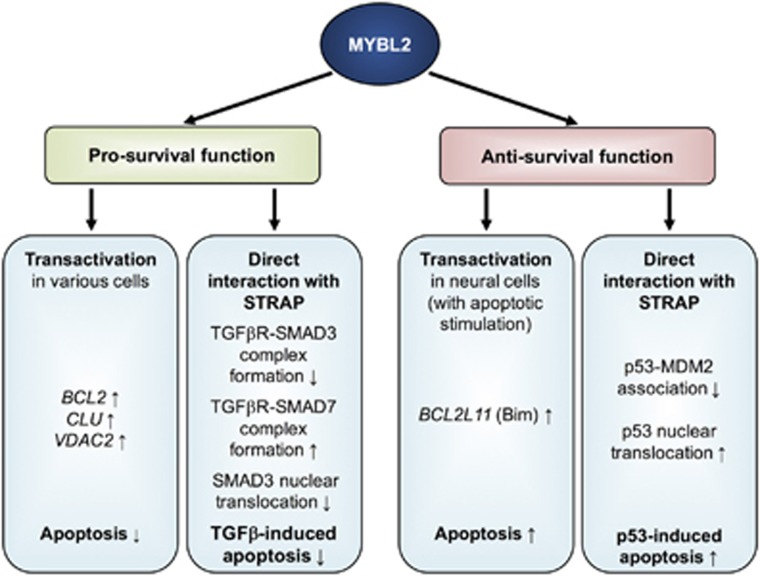
MYBL2 in regulation of cell survival. MYBL2 is primarily described to have pro-survival functions, but also anti-survival functions of MYBL2 were shown. These effects are mainly mediated by MYBL2 transactivating target genes regulating cell survival (pro-survival: *BCL2*, *CLU*, *VDAC*; anti-survival: *BCL2L11* (Bim)). Pro-survival functions were described in various cell types, whereas anti-survival functions were mainly described in cells of neural origin when exposed to apoptotic stimuli. Apart from transactivation of its target genes, MYBL2 can also regulate cell survival by direct protein–protein interaction with STRAP. On the one hand, this can lead to inhibition of TGF-*β* signaling pathways by inhibiting TGF-*β* receptor association with SMAD3 and enhancing TGF-*β* receptor association with SMAD7, and thereby prevent translocation of SMAD3 in the nucleus in response to TGF-*β*1 (pro-survival function). On the other hand, it can result in increased STRAP-mediated stimulation of p53 nuclear translocation, p53-induced apoptosis and cell cycle arrest via reduction of p53-MDM2 association

An association between MYBL2 and cell survival has already been reported in early studies. However, over the years, the role of MYBL2 in the regulation of cell survival became increasingly clear and is mainly mediated via transcriptional regulation of specific target genes, but can also be mediated by direct protein–protein interaction. The transcriptional regulation by MYBL2 seems to depend on the cell type: In most cell types MYBL2 appears to have pro-survival functions, whereas it mainly has anti-survival functions in cells of neural origin when exposed to apoptotic stimuli (Figure 2).

### Pro-survival function via transcriptional regulation

Grassilli *et al.*^47^ showed that MYBL2 overexpression in interleukin 2-dependent murine T cells is associated with enhanced transactivation of the anti-apoptotic Bcl-2, and hence diminished cytokine dependence and enhanced resistance to apoptosis induced by doxorubicin, ceramide and dexamethasone. Consistently, the transfection of a Bcl-2-non-expressing human B-cell line with a *MYBL2* expression vector induced the expression of Bcl-2 and vice versa, antisense depletion of MYBL2 decreases Bcl-2 levels and enhances apoptosis.^70^ Furthermore, results of Cervellera *et al.*^50^ indicate that *ApolipoproteinJ/Clusterin* is a MYBL2 target gene, whose expression mediates resistance to apoptosis induced by the chemotherapeutic drug doxorubicin in neuroblastoma. Santilli *et al.*^62^ further confirm these results: under conditions of thermal stress, MYBL2-dependent *ApolipoproteinJ*/*Clusterin* expression is enhanced due to redox modification of MYBL2 and constitutes a protective response mechanism to thermal injury in MEFs. MYBL2 has also been shown to suppress autophagy and to promote cell survival of ovarian oocytes by binding the promoter and directly activating the transcription of *VDAC2*.^51^

### Anti-survival function via transcriptional regulation

However, in contrast to these findings, MYBL2 seems to have a contrary role concerning cell survival predominantly in neural cells. MYBL2 knockdown protects pheochromocytoma cells, sympathetic neurons and cortical neurons against cell death elicited by NGF withdrawal or DNA damage.^71^ This indicates a required role for MYBL2 in neuronal apoptosis after E2F de-repression due to apoptotic stimuli.^71, 72^ A model has been proposed by which E2F4–p130 protein complexes protect neurons from cell death by occupying the *MYBL2* promoter under basal conditions, whereas under conditions of cell stress these complexes are lost and replaced by E2F1 transactivating *MYBL2* and thus promoting cell death.^73^ In neurons, MYBL2 is able to bind the promoter and to transactivate the pro-apoptotic gene *BCL2L11* (Bim).^48^ The following interaction of Bim with the cellular apoptotic machinery leads to caspase activation and apoptotic cell death.^48^ MYBL2 was also shown to be required for beta-amyloid-dependent induction of Bim and cell death, relevant in Alzheimer’s disease.^74^ Also in *Drosophila*, dMyb promotes the programmed cell death of specified sensory organ precursor daughter cells.^75^ However, not only in neural cells, but also in TGF-*β*1-treated M1 myeloid leukemia cell lines overexpressing MYBL2, TGF-*β*1-induced apoptosis was found to be accelerated.^76^

### Direct protein–protein interactions

Independent of the transactivation capabilities of MYBL2, it is further able to regulate cell survival by direct interaction with the serine–threonine kinase receptor-associated protein (STRAP), for which MYBL2 can serve as a positive regulator.^77^ On the one hand, MYBL2 can enhance STRAP-mediated inhibition of TGF-*β* signaling pathways, such as apoptosis and growth inhibition, by inhibiting TGF-*β* receptor association with SMAD3 and enhancing TGF-*β* receptor association with SMAD7, and thereby prevent translocation of SMAD3 in the nucleus in response to TGF-*β*1 (pro-survival function).^77^ On the other hand, co-expression of MYBL2 results in increased STRAP-mediated stimulation of p53 nuclear translocation, p53-induced apoptosis and cell cycle arrest via reduction of p53–MDM2 association (anti-survival function).^77^

## MYBL2 in differentiation and maintenance of stem cell properties

Several lines of evidence indicate that MYBL2 contributes to the maintenance of an undifferentiated and/or stem cell-like phenotype of a cell. Especially in stem cells, the balance between cellular quiescence on the one hand, and cell division in order to generate more stem cells (self-renewal) or to give rise to mature cells (differentiation) on the other hand, is important for the maintenance of the stem cell pool.^78^

Early results showed that MYBL2 protein levels decrease upon differentiation of human myeloid cell lines.^79^ Later on, in neuroblastoma cells, MYBL2 expression was found to be downregulated during retinoic acid-induced neural and glial differentiation and conversely, constitutive expression of MYBL2 prevents retinoic acid-induced neural differentiation.^80^ Compatible with this, levels of p130, a member of the DREAM complex (see ‘MYBL2 in cell cycle regulation’ section) that is able to suppress the *MYBL2* promoter upon transfection, was shown to be strongly upregulated during mid/late differentiation stages, whereas MYBL2 levels decrease.^81^ Comparable results indicating a role for MYBL2 to maintain cells in an undifferentiated state have also been shown for several different cell types, such as leukemic cell lines,^82^ male gonocytes,^83^ intestinal epithelial cells^38^ and keratinocytes.^84^

**Figure 3:**
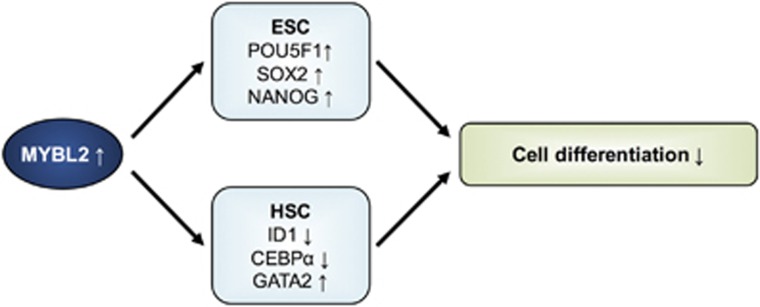
MYBL2 in differentiation and maintenance of stem cell properties. In various studies, high MYBL2 levels were shown to be associated with cell dedifferentiation (see ‘MYBL2 in differentiation and maintenance of stem cell properties’ section). Mechanistically, the role of MYBL2 in regulation of differentiation was mainly investigated in embryonic stem cells (ESC) and hematopoietic stem cells (HSC): In ESC, MYBL2 was shown to directly control the expression of *POU5F1*, *SOX2* and *NANOG*, which are critical regulators of differentiation and maintenance of pluripotency. In HSC, MYBL2 was shown to downregulate *ID1* and *CEBPα*, which promote cellular differentiation, and to upregulate *GATA2*, a transcription factor shown to promote proliferation at the expense of differentiation. Thus, MYBL2 helps the cell to maintain in an undifferentiated, pluripotent, but proliferative state

Mechanistically, for the maintenance of a pluripotent and undifferentiated phenotype of ESC, it was proposed that MYBL2 may regulate a transcriptional network that controls cell cycle progression and cell fate to sustain self-renewal and pluripotency.^52^ Especially for the maintenance of pluripotency, MYBL2 directly regulates the expression of *POU5F1, SOX2* and *NANOG*, which are critical mediators of differentiation and pluripotency in ESC.^52, 53, 85^ Similarly, MYBL2 was shown to control self-renewal and differentiation of hematopoietic stem cells, possibly by downregulating *ID1* and *CEBPα*, which promote cellular differentiation, while upregulating *GATA2,* a transcription factor shown to promote proliferation at the expense of differentiation^78, 86^(Figure 3).

In summary, these studies indicate that MYBL2 helps the cell to maintain an undifferentiated, pluripotent, but proliferative state.

## MYBL2 in Cancer

**Figure 4:**
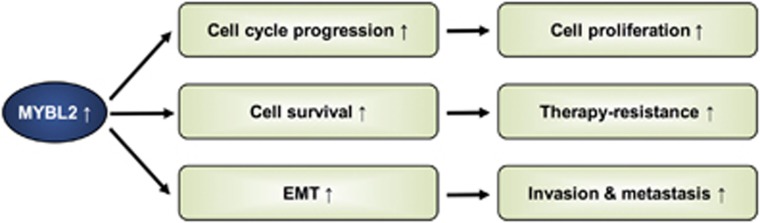
MYBL2 deregulation in promotion of cancer initiation and/or progression. Upregulation of MYBL2 is described in numerous cancer entities and is associated with poor patient outcome (Table 2). It leads to an increase in cell cycle progression, cell survival and epithelial-to-mesenchymal transition (EMT), thus promoting cell proliferation, therapy resistance, invasion and metastatic spread

The roles of MYBL2 in cell cycle progression, cell survival and cell differentiation suggest that deregulation of MYBL2 may has an oncogenic potential. It can contribute significantly to cancer progression by promoting cancer cell proliferation, therapy resistance and metastatic spread (Figure 4). Indeed, MYBL2 is frequently found to be overexpressed in several cancer entities and associated with poor patient outcome^87, 88, 89, 90, 91, 92, 93^ (Table 2).

### Mechanisms of MYBL2 deregulation in cancer

On the one hand, altered *MYBL2* expression can arise from chr20q13 amplification, which is described for several cancer entities, for example, breast cancer, colorectal cancer and ovarian cancer.^94, 95, 96^ On the other hand, it can be caused by deregulation of DREAM complex assembly, for example, due to p53 mutation or transformation by the HPV16 E7 oncogene and thereby uncoupling *MYBL2* expression from negative transcriptional regulation and enabling MYBL2 to increasingly bind to MuvB and FOXM1 (see below). Additionally, MYBL2 expression can be deregulated at a post-transcriptional level via deregulation of microRNAs, a class of small non-coding RNAs often found to be deregulated in cancer^97^ and of which some were shown to suppress MYBL2 mRNA expression.^14, 15, 16, 17, 18, 19^

p53 signaling is frequently altered in cancers.^98^ Physiologically, p53 directly activates p21, which prevents following p130 phosphorylation by cyclin-dependent kinases, leading to a switch from MYBL2–MuvB to DREAM complex by shifting MuvB-associated proteins from MYBL2 to E2F4/DP1/p130.^99^ It has been described that the p53-p21-DREAM pathway represses MYBL2 expression, especially under conditions of cellular stress, such as DNA damage, which mechanistically explains MYBL2 deregulation, and thus deregulation of cell cycle progression and cell survival in p53-mutated cancers.^100, 101^ This is in accordance with results from Parikh *et al.*^102^ showing that MYBL2 is disproportionately upregulated in many p53 mutant cancers. MYBL2 has even been shown to overcome DNA damage-induced G2 checkpoint arrest in p53 mutant cells^103^ and constitutive expression of MYBL2 has been shown to overcome p53-induced G1 checkpoint arrest.^59^ Furthermore, the oncoviral HPV16 E7 protein is able to deregulate DREAM complex assembly and to thereby drive MYBL2 expression.^104^ Consistent to this, HPV16-immortalized cells show upregulated expression of MYBL2.^105^ Mechanistically, the HPV16 E7 oncogene can bind to p130, promote its proteasomal degradation and thereby disassemble the DREAM complex.^106^ E7 in addition directly binds to the MYBL2–MuvB–FoxM1 complex, leading to cooperative transcriptional activation of mitotic genes.^106^ MYBL2 moreover mediates abrogation of DNA damage-induced G1 checkpoint arrest, via regulation of CDK1 expression in E7 transformed cells^107^ and was shown to rescue oncogene-induced cellular senescence,^14, 108^ a permanent cell cycle arrest that cells must bypass during cancer development,^14, 109^ probably by suppressing p16^INK4A^ expression.^14, 108, 110^

### MYBL2 in deregulation of proliferation

As described for non-malignant cells (see ‘MYBL2 in cell cycle regulation’ section), MYBL2 has also been shown to drive cell proliferation and/or cell cycle progression in cancer cells, such as breast cancer,^111^ cervical cancer,^112^ colorectal cancer,^89^ hepatocellular carcinoma,^91^ leukemic cells,^15^ lung adenocarcinoma^42^ and neuroblastoma (in MYCN-amplified cell lines).^113^

### MYBL2 in cancer therapy resistance

Resistance to chemo- and radiotherapy is one of the main properties of a cancer that determines cancer progression and patient outcome. MYBL2 overexpression in interleukin 2-dependent murine T cells was shown to be associated with enhanced resistance to drug-induced apoptosis by doxorubicin, ceramide and dexamethasone, due to increased transactivation of the anti-apoptotic Bcl-2 by MYBL2.^47^ These results from Grassilli *et al.* are in accordance with results from Levenson *et al.,*^114^ showing that MYBL2 is upregulated upon genetic suppressor element-induced drug resistance to DNA-interactive agents, such as aphidicolin, hydroxyurea, cytarabine, etoposide, doxorubicin and mafosfamide in fibrosarcoma cells. In neuroblastoma, MYBL2 directly regulates expression of ApolipoproteinJ/Clusterin and thereby mediates resistance to apoptosis induced by doxorubicin.^50^

However, MYBL2 was not only shown to mediate resistance to chemotherapeutic agents, but also resistance to DNA damage, as, for example, caused by radiation. Under physiological conditions, such as in p53 wild-type cells, DNA damage results in p53-dependent binding of p130 and E2F4 to MuvB and the dissociation of the MYBL2–MuvB complex.^99, 100, 101^ Also, upon ionizing radiation, Cyclin F suppresses the MYBL2-regulated transcriptional program by directly interacting with MYBL2 and thereby suppressing Cyclin A-mediated phosphorylation of MYBL2.^115^ On the contrary, under non-physiological conditions, such as in p53 mutant cells, MYBL2 fails to dissociate from MuvB, which contributes to increased G2/M gene expression in response to DNA damage.^103^ In accordance, DT40 chicken B cells lacking MYBL2 show increased sensitivity to DNA damage elicited by UV irradiation and alkylation.^116^ Consistently, in Ewing sarcoma cells, MYBL2 can be destroyed quickly upon UV irradiation, leading to induction of apoptosis,^117^ whereas this is not the case in neuroblastoma, where MYBL2 levels do not change upon irradiation, making the cells resistant to UV-induced apoptosis.^117^ Interestingly, in neuroblastoma cells MYBL2 is found to be hypophosphorylated and overexpression of a non-phosphorylatable MYBL2 mutant in HEK 293 cells can protect cells from UV-induced apoptotic cell death, suggesting that decreased Cyclin A-dependent phosphorylation, accompanied by decreased activation but also decreased proteasomal degradation, can facilitate the survival promoting activity of MYBL2.^117^

Consistent with these results, a pro-survival role for MYBL2 has also been shown in several cancer cell lines, such as colorectal cancer,^89^ hepatocellular carcinoma^91^ and leukemia cells.^118^

### MYBL2 in invasion and metastasis

Early results of Iwai *et al.*^58^ have shown that the introduction of an inducible dominant interfering Myb protein into ESC lead to dissociation of ESC colonies into dispersed single cells and to reduced adhesion of the ESC to the culture dish. Cell adhesion analyses have shown that MYBL2 suppression decreased the adhesion with extracellular matrix proteins, such as Laminin, Collagen and Fibronectin, probably due to reduced cell surface expression of Beta1 Integrin.^58^

However, in contrast to these early findings, a role for MYBL2 in epithelial-to-mesenchymal transition (EMT), a process in which epithelial cells lose their polarity and gain migratory and invasive properties,^119^ has been proposed: In breast cancer cells, MYBL2 knockdown is able to restore the expression of the epithelial marker E-Cadherin, the formation of cell–cell junctions and to suppress cell invasion, anchorage-independent growth and tumor formation.^120^ Conversely, MYBL2 overexpression decreased the expression of the E-Cadherin, but increased expression of mesenchymal markers.^120^ Mechanistically, it was proposed that MYBL2 upregulates the expression of the major EMT regulator SNAIL, thereby mediating activation of EMT and cancer cell invasion.^120^

In accordance with this, MYBL2 protein levels have been shown to be significantly upregulated in matched breast cancer metastases compared to the primary tumor.^111^ Similar results were shown for prostate cancer and renal cell carcinoma: MYBL2 is overexpressed in prostate cancer (xenograft) metastases,^121^ whereas in renal cell carcinoma MYBL2 was found to be expressed in metastases from primary tumors being MYBL2 negative.^93^

## Conclusions

MYBL2 is a central regulator of cell cycle progression, cell survival and cell differentiation. Deregulation of MYBL2 expression can contribute significantly to cancer progression by promoting cancer cell proliferation, therapy resistance, metastatic spread and is correlated with poor patient outcome in several cancer entities. Therefore, MYBL2 and/or players of its downstream transcriptional network could serve as effective targets for cancer treatment. Although no direct MYBL2 inhibitor is available yet, CDK2 inhibition could be used to reduce MYBL2 activity in MYBL2 high-expressing cancers. Yet, highly specific CDK2 inhibitors are lacking, but several more or less specific CDK inhibitors have already been in clinical trials for cancer treatment.^122, 123^ Also, several inhibitors interfering with players of the downstream transcriptional network of MYBL2, such as inhibitors against Aurora kinases,^124^ FGF receptors,^125^ Kinesins,^126^ Bcl-2^127^ and BIRC5 (Survivin)^128^ have already been in clinical trials and may serve as an effective, more specific and less toxic future anti-cancer therapy in cancers highly expressing MYBL2.

## Figures and Tables

**Table 1:** Selected target genes transactivated by MYBL2

**Gene symbol**	**Protein name**	**Reference(s)**
*Cell cycle regulation*		
AURKA	Aurora A kinase	Sadasivam *et al.*^36^
CCNA1 (Sp1-mediated)	Cyclin A1	Bartusel *et al.*^35^
CCNA2	Cyclin A2	Zhu *et al.*^34^; Osterloh *et al.*^37^
CCNB1	Cyclin B1	Zhu *et al.*^34^; Sadasivam *et al.*^36^; Osterloh *et al.*^37^
CCND1 (Sp1-mediated)	Cyclin D1	Bartusel *et al.*^35^
CCND2 (repression)	Cyclin D2	Papetti *et al.*^38^
CDK1	Cyclin-dependent kinase 1	Zhu *et al.*^34^; Osterloh *et al.*^37^
CDK2 (repression)	Cyclin-dependent kinase 2	Papetti *et al.*^38^
CDKN2A (repression)	p16^INK4A^	Huang *et al.*^110^
CENPF	Centromere protein F	Iltzsche *et al.*^42^
CEP55	Centrosomal protein 55	Wolter *et al.*^43^
FGF4	Fibroblast growth factor 4	Johnson *et al.*^40^
FOXM1	Forkhead box M1	Lorvellec *et al.*^41^
KIFC1; KIF2C; KIF4A; KIF14; KIF20A; KIF23	Mitotic kinesins	Wolter *et al.*^43^; Iltzsche *et al.*^42^
MYB (repression)	c-Myb	Guerra *et al.*^44^
MYBL2 (Sp1-mediated)	B-Myb	Sala *et al.*^45^
MYC	c-Myc	Nakagoshi *et al.*^46^ (activation); Lorvellec *et al.*^41^ (activation); Papetti *et al.*^38^ (repression)
NUSAP1	Nuclear- and spindle-associated protein 1	Iltzsche *et al.*^42^
PLK1	Polo-like kinase 1	Sadasivam *et al.*^36^; Osterloh *et al.*^37^
PRC1	Protein regulator of cytokinesis 1	Wolter *et al.*^43^
TOP2A	DNA topoisomerase II*α*	Brandt *et al.*^39^
		
*Cell survival*		
BCL2	Bcl-2	Grassilli *et al.*^47^
BCL2L11	Bim	Greene *et al.*^48^
BIRC5	Survivin	Knight *et al.*^49^
CLU	ApolipoproteinJ/Clusterin	Cervellera *et al.*^50^
FGF4	Fibroblast growth factor 4	Johnson *et al.*^40^
MYB (repression)	c-Myb	Guerra *et al.*^44^
MYBL2 (Sp1-mediated)	B-Myb	Sala *et al.*^45^
MYC	c-Myc	Nakagoshi *et al.*^46^ (activation); Lorvellec *et al.*^41^ (activation); Papetti *et al.*^38^ (repression)
PLK1	Polo-like kinase 1	Sadasivam *et al.*^36^; Osterloh *et al.*^37^
VDAC2	Voltage-dependent anion channel 2	Yuan *et al.*^51^
		
*Differentiation*		
NANOG	Homeobox protein NANOG	Zhan *et al.*^52^
POU5F1	Oct-4	Tarasov *et al.*^53^
SOX2	Sox2	Zhan *et al.*^52^
		
*Invasion/metastasis*		
SNAI1	Snail (Zinc-finger protein SNAI1)	Tao *et al.*^120^

**Table 2:** MYBL2 deregulation associated with patient outcome in different cancer entities

**Tumor entity**	**MYBL2 deregulation**	**Association with patient survival**	**References**
Acute myeloid leukemia	Overexpression	MYBL2 expression is an independent prognostic factor for disease-free survival and cumulative incidence of relapse	Fuster *et al.*^17^
Bladder carcinoma	Overexpression (amplification)	Overrepresentation of amplicons in high-grade and recurrent cases	Nord *et al.*^87^
Breast cancer	Overexpression	Overexpression is associated with short overall patient survival and short disease-free survival	Inoue and Fry^88^
Colorectal cancer	Overexpression	Overexpression is correlated with worse disease-free survival and MYBL2 is an independent prognostic factor for poor patient survival	Ren *et al.*^89^
Esophageal squamous cell carcinoma	Overexpression (amplification)	High MYBL2 expression and high MYBL2 copy-number are associated with poor patient survival	Qin *et al.*^90^
Hepatocellular carcinoma	Overexpression, high phosphorylation levels	High levels of total and phosphorylated MYBL2 and high levels of LIN9–MYBL2 complex in HCC with poorer outcome	Calvisi *et al.*^91^
Neuroblastoma	Overexpression	MYBL2 expression is associated with increased risk of death and worse overall survival	Raschellà *et al.*^92^
Renal cell carcinoma	Positivity	Positivity for MYBL2 expression is significantly correlated with clinical stage III and IV	Sakai *et al.*^93^
